# Acetylcorynoline Induces Apoptosis and G2/M Phase Arrest through the c-Myc Signaling Pathway in Colon Cancer Cells

**DOI:** 10.3390/ijms242417589

**Published:** 2023-12-18

**Authors:** Ye-Rin Park, Wona Jee, So-Mi Park, Seok-Woo Kim, Ji-Hoon Jung, Hyungsuk Kim, Kwan-Il Kim, Hyeung-Jin Jang

**Affiliations:** 1 College of Korean Medicine, Kyung Hee University, 24, Kyungheedae-ro, Dongdaemun-gu, Seoul 02447, Republic of Korea; yerinp@khu.ac.kr (Y.-R.P.); jee1ah@khu.ac.kr (W.J.); psm991030@naver.com (S.-M.P.); kim66470@naver.com (S.-W.K.); johnsperfume@gmail.com (J.-H.J.); kim0874@hanmail.net (H.K.); kwanilkim@khu.ac.kr (K.-I.K.); 2 Department of Science in Korean Medicine, Graduate School, Kyung Hee University, Seoul 02447, Republic of Korea; 3 Department of Korean Rehabilitation Medicine, Kyung Hee University Medical Center, Seoul 02447, Republic of Korea; 4 Division of Allergy, Immune and Respiratory System, Department of Internal Medicine, College of Korean Medicine, Kyung Hee Medical Center, Kyung Hee University, Seoul 02447, Republic of Korea

**Keywords:** acetylcorynoline, c-Myc, CNOT2, MID1IP1, colon cancer

## Abstract

Colorectal cancer (CRC) is the third most commonly diagnosed cancer worldwide, and despite advances in treatment, survival rates are still low; therefore, the development of novel drugs is imperative. Acetylcorynoline (ACN) is derived from *Corydalis ambigua Cham. et Schltdl* tubers. The effect of ACN on colon cancer is still unknown. Therefore, we investigated its potential effects. Our data showed that ACN inhibited cell viability and proliferation. Moreover, ACN induced apoptosis and cell cycle arrest by inhibiting cell growth. In the present study, we hypothesized that ACN regulates c-Myc through CNOT2 or MID1IP1. ACN reduced the protein expression of oncogenic genes, decreased c-Myc half-life, and rapidly inhibited the serum stimulation response. Moreover, knockdown of CNOT2 and MID1IP1 with ACN increased apoptosis and further reduced the expression of oncogenes. In addition, ACN exhibited a synergistic effect with low-dose 5-fluorouracil (5-FU) and doxorubicin (Dox). Collectively, our data demonstrate that ACN inhibited c-Myc expression through CNOT2 and MID1IP1, and induced apoptosis. These findings indicate the potential of ACN as a therapeutic agent against colon cancer.

## 1. Introduction

Colorectal cancer (CRC) is the third most commonly diagnosed cancer worldwide. CRC constitutes 10.0% of the most commonly diagnosed cancers and accounts for 9.4% of cancer-related deaths [1]. Despite recent advances in treatment, only 39% of CRC cases are diagnosed at the local stage; the remaining patients are diagnosed at the regional or distant stages, where survival rates are significantly reduced [2,3]. Currently, 5-Fluorouracil (5-FU) and doxorubicin (Dox) are primarily prescribed as chemotherapeutic drugs for patients with CRC [4], and the 5-year survival rate is still low because of dose-limiting toxicity and drug resistance when administered for a long time. Therefore, the development of novel and effective adjuvant treatments that minimize the side effects in CRC is urgently required.

To confront this challenge, research has been conducted on natural product-derived compounds with comparatively low toxicity, aiming to enhance the effectiveness of chemotherapy such as 5-FU and Dox while minimizing side effects. Additionally, utilizing compounds derived from natural products to target specific oncogenes such as c-Myc, CCR4-NOT transcription complex subunit 2 (CNOT2) and MID1-interacting protein 1 (MID1IP1) have been proposed as an innovative treatment for various cancers, particularly CRC [5,6,7].

c-Myc, a member of the proto-oncogenic transcription factor family, is involved in regulating cell growth and proliferation in normal cells. Uncontrolled c-Myc overexpression contributes to most cancers, including colon cancer [8,9]. Controlling the c-Myc signaling pathway within cells can be a treatment strategy for various types of cancer by inducing apoptosis and inhibiting proliferation [10]. Therefore, reducing c-Myc overexpression can inhibit the progression of colon cancer.

CNOT2 plays a crucial role in apoptosis, autophagy, proliferation, and angiogenesis in cancer cells. Previous studies have shown that the depletion of CNOT2 can increase apoptosis and decrease metastasis through proliferation and inhibition of angiogenesis [11,12]. MID1IP1, also known as MIG12, is highly expressed in various cancer types. A recent study has shown that MID1IP1 acts upstream of AMP-activated protein kinase (AMPK) and regulates AMPK phosphorylation in liver cancer cells [13]. Additionally, MID1IP1 contributes to cancer cell growth and death through co-localization with c-Myc-mediated ribosomal proteins L5, L11, and CNOT2 [6].

Corydalis species are one of traditional Chinese medicines for pain relief, known for their anti-inflammatory and anticancer effects [14,15]. In traditional Chinese medicine, this substance is utilized for the treatment of various ailments, including abdominal pain, menstrual cramps, and bleeding hemorrhoids. Acetylcorynoline (ACN) is a major alkaloid derived from *C. ambigua Cham. et Schltdl* tubers. ACN has been shown to be effective in the treatment of Parkinson’s disease (PD) [16], reduce carbon tetrachloride (CCl4)-induced microsomal lipid peroxidation [17], and is anti-inflammatory [18]. However, the anticancer effects on colon cancer cells and molecular mechanisms of action of ACN remain insufficient in colon cancer.

In conclusion, our study aims to systematically explore of the potential anticancer effects of ACN in colon cancer cells. We particularly investigate how ACN induces anticancer effects through mechanisms involving c-Myc, CNOT2, and MID1IP1. Through a comprehensive exploration of these interactions, we anticipate providing novel insights into the therapeutic potential of ACN for CRC.

## 2. Results

### 2.1. ACN Inhibits the Cell Viability and Proliferation of Colon Cancer Cells

**Figure 1:**
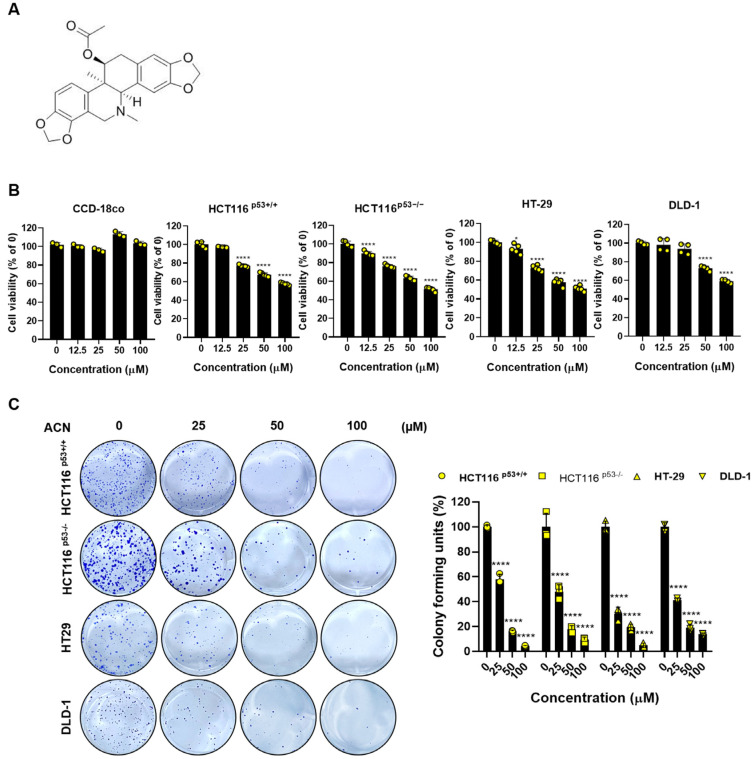
Cell viability and proliferation effects of ACN in colon cancer cells. (**A**) The structure of ACN. (**B**) The effects of ACN (25–100 µM) on the cell viability of non-cancerous CCD-18co and colon cancer cells shown using the MTT assay. (**C**) Colony formation ability and quantification of colony forming unit percentage. Data (n ≥ 3) are represented as the mean ± SD. * *p* < 0.1. **** *p* < 0.0001.

To investigate the effects of ACN, we performed biological assessments on four types of colon cancer cells: HCT116^p53+/+^, HCT116^p53−/−^, HT-29, and DLD-1. Cell viability was determined using an MTT assay. ACN inhibited the colon cancer cell viability in a dose-dependent manner, whereas the non-cancerous colon cell line CCD-18co did not affect the cell viability when treated with up to 100 µM (Figure 1B). In addition, the colony formation assay showed that ACN inhibited cell proliferation compared to the untreated group (Figure 1C). In conclusion, our data indicate that ACN is involved in the viability and proliferation of colon cancer cells.

### 2.2. ACN Regulates Apoptosis and Induces G2/M Phase Arrest

**Figure 2:**
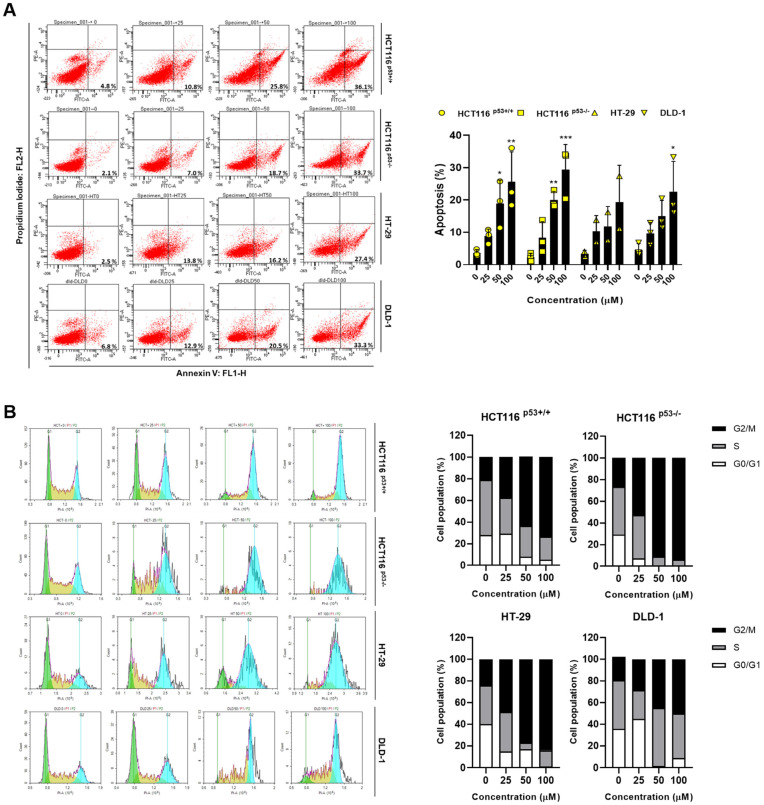
ACN induces apoptosis and cell cycle arrest. (**A**) Apoptosis analysis using Annexin V/PI staining with flow cytometry. (**B**) Cell cycle analysis using flow cytometry. Data (n ≥ 3) are represented as the mean ± SD. * *p* < 0.05, ** *p* <0.01, and *** *p* < 0.001.

To demonstrate the ACN-induced inhibition of colon cancer cell growth, we performed flow cytometry. Then, we performed annexin V and PI staining to assess whether ACN imparts cell death via apoptosis. As shown in Figure 2A, apoptosis rates were significantly increased in ACN-treated colon cancer cells compared to those in the untreated group. We also investigated the effect of ACN on cell cycle distribution. Colon cancer cells were exposed to each concentration of ACN for 24 h and stained with PI, followed by an analysis of the cell cycle pattern. ACN increased the population of the G2/M phase (Figure 2B), indicating that ACN induces apoptosis via cell cycle arrest.

### 2.3. ACN Regulates the Expression of Apoptosis-Related Signaling Proteins

**Figure 3:**
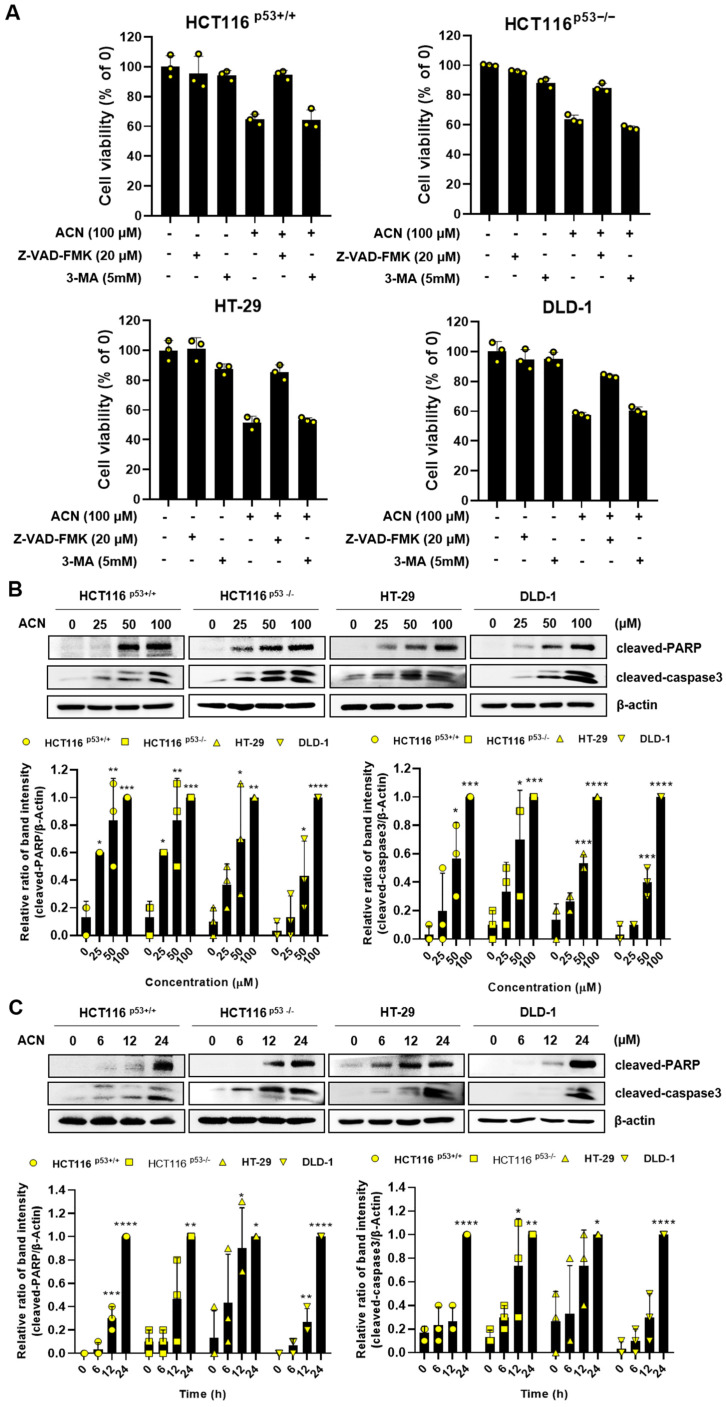
ACN leads to apoptosis in colon cancer cells. (**A**) The MTT assay was conducted via treatment with ACN, apoptosis inhibitor (20 μM, Z-VAD-FMK), and autophagy inhibitor (5 mM, 3-MA). The protein expressions were investigated using Western blotting in a dose- (**B**) and time- (**C**) dependent manner. Data (n ≥ 3) are represented as the mean ± SD. * *p* < 0.05, ** *p* <0.01, *** *p* < 0.001, and **** *p* < 0.0001.

To investigate the type of cell death, we exposed ACN to the apoptosis inhibitor (Z-VAD-FMK) or autophagy inhibitor (3-methyladenine, 3-MA). The apoptosis inhibitor effectively rescued ACN-induced cell death, whereas the autophagy inhibitor did not (Figure 3A), indicating that ACN might induce apoptosis. Cysteine-dependent aspartate-specific proteases (caspase), as one of the most common pathways in apoptosis, induce cell death by cleaving and activating effector caspases upon their activation, thereby triggering apoptosis [19]. The function of poly (ADP-ribose) polymerase (PARP) by caspases is recognized as a hallmark of apoptosis, and PARP stands among various cellular substrates targeted by caspases [20]. We confirmed the induction of apoptosis by ACN through annexin V staining. Therefore, we investigated the expression of apoptosis-related proteins. ACN treatment increased the expression of cleaved PARP and cleaved caspase3 was increased in a dose- and time-dependent manner (Figure 3A,B). In conclusion, we have demonstrated that ACN-induced apoptosis occurred through the upregulation of key regulators of apoptosis, cleaved PARP and cleaved caspase3.

### 2.4. c-Myc Is a Potential Target for the Inhibition of Colon Cancer in ACN

**Figure 4:**
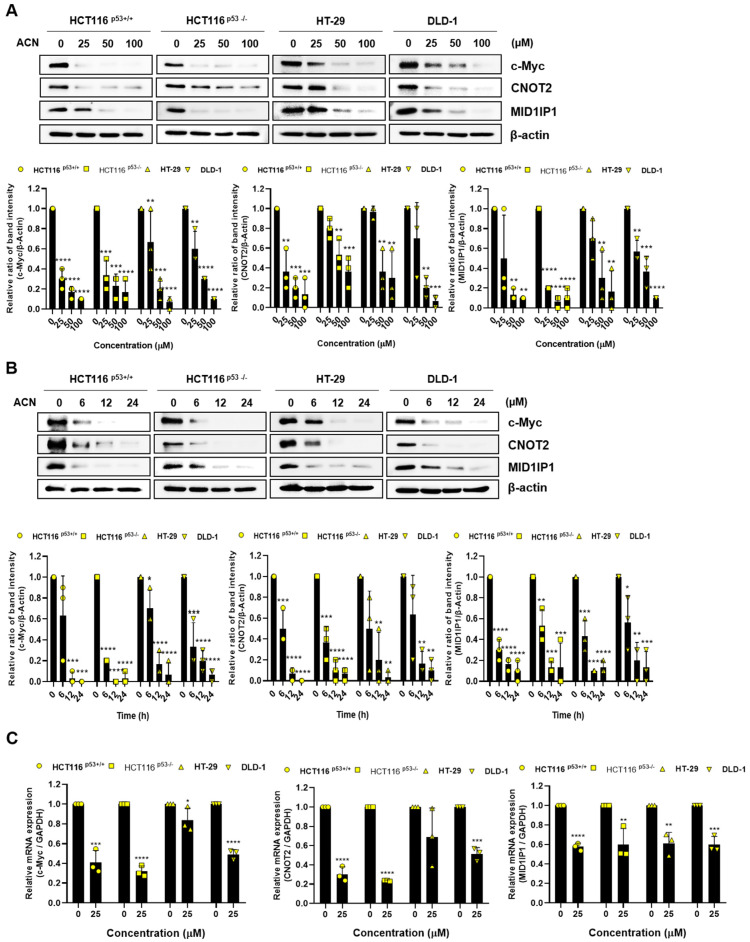

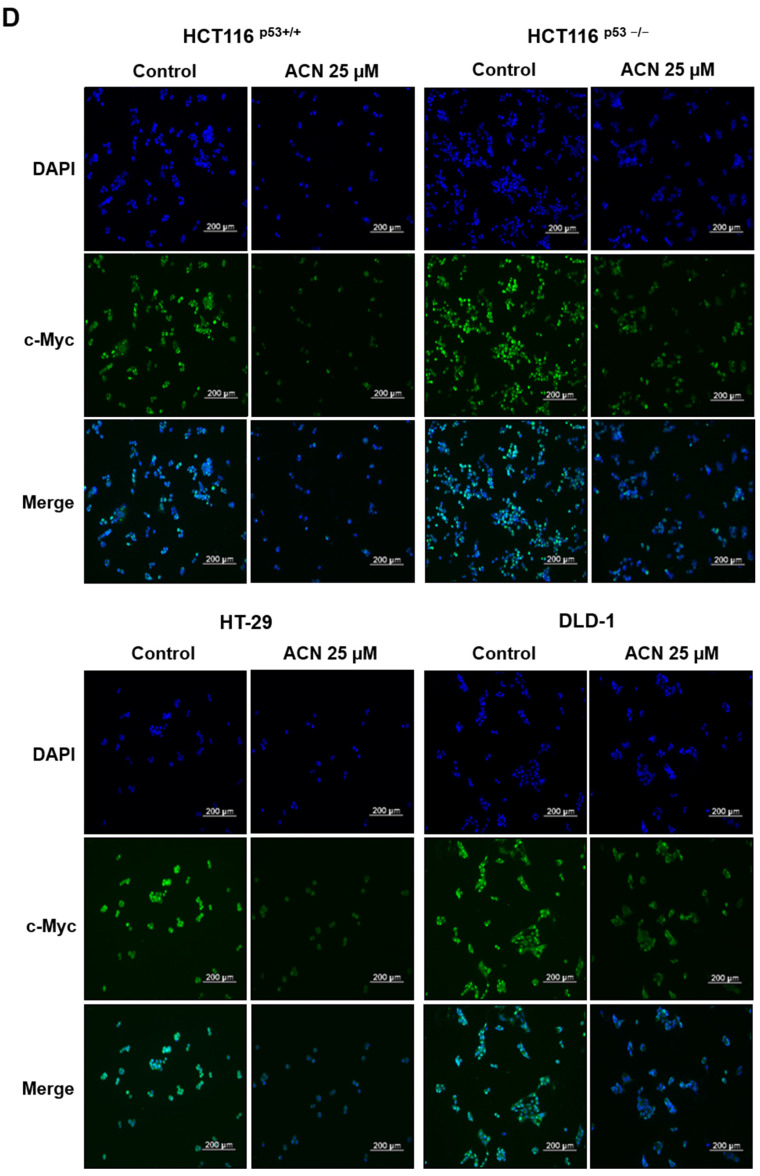
Downregulation of c-Myc through the CNOT2 and MID1IP1 of ACN. ACN regulates the protein expression of oncogenes in a (**A**) dose- and (**B**) time-dependent manner. (**C**) mRNA expression after treatment with ACN. (**D**) Decreased fluorescence of c-Myc in ACN-treated colon cancer cells. Data (n ≥ 3) are represented as the mean ± SD. * *p* < 0.05, ** *p* <0.01, *** *p* < 0.001, and **** *p* < 0.0001.

c-Myc is a transcriptional regulator that controls the expression of various genes and regulates proliferation, the cell cycle, and the survival of cancer cells [21,22]. MID1IP1, which induces apoptosis and is controlled by CNOT2, regulates c-Myc via ribosomal proteins L5 and L11, and is involved in cancer cell growth [6,23]. c-Myc is overexpressed in CRC and plays a potential role in colon cancer development [24]. We hypothesized that the effects of ACN-induced apoptosis and cell cycle arrest may be related to c-Myc signaling. To investigate the ACN regulation of c-Myc, CNOT2, and MID1IP1 protein expression level, immunoblotting was performed. The protein expression of c-Myc, CNOT2, and MID1IP1 decreased in a dose- and time-dependent manner after ACN treatment of colon cancer cells (Figure 4A,B). Further, we investigated the mRNA expression of c-MYC, CNOT2, and MID1IP1 using real-time PCR (Figure 4C). ACN significantly reduced mRNA levels of c-Myc, CNOT2, and MID1IP1. Immunofluorescence staining was performed to confirm the c-Myc expression. The results showed a decrease in c-Myc expression after ACN treatment (Figure 4D). In this figure, we have demonstrated that ACN inhibits cancer cell survival and proliferation by regulating c-Myc.

### 2.5. ACN Attenuates c-Myc via CNOT2, MID1IP1, and Ribosomal Proteins

**Figure 5:**
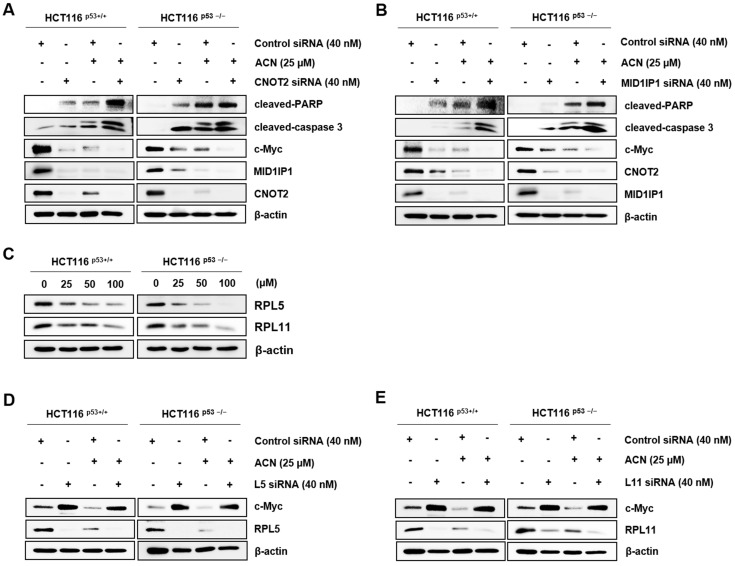
The effect of ribosomal protein L5 or L11 on c-Myc expression in ACN treatment. (**A**,**B**) Apoptosis upregulation via CNOT2 and MID1IP1 depletion. (**C**) The effect of ACN on RPL5 and RPL11. (**D**,**E**) Regulation of c-Myc through ribosomal proteins L5 and L11 in ACN treatment. Data (n ≥ 3) are represented as the mean ± SD.

To confirm whether ACN is related to c-Myc through CNOT2 and MID1IP1, we knocked down CNOT2 and MID1IP1 using siRNA with or without ACN. As shown in Figure 5A,B, ACN further reduced c-Myc protein expression via CNOT2 and MID1IP1 depletion and strengthened apoptosis by increasing cleaved PARP and cleaved caspase3. Our results suggest that the reduction in MID1IP1, which co-localizes with c-Myc, is mediated by ribosomal proteins L5 and L11. Therefore, we investigated whether ACN influences L5 and L11 [6]. Additionally, the depletion of RPL5 and RPL11 regulates cell proliferation and cell cycle [25]. Thus, we have examined whether ACN influences ribosomal proteins, and we demonstrated that ACN inhibits RPL5 and RPL11 (Figure 5C). ACN reduced the expression of MID1IP1 and c-Myc; simultaneously, the loss of L5 and L11 reversed the expression of c-Myc reduction to ACN in HCT116^p53+/+^ and HCT116^p53−/−^, which showed the least expression of c-Myc at 25 µM in four CRC cell lines (Figure 5D,E). Collectively, our results demonstrate that the regulation of c-Myc via CNOT2, MID1IP1, and ribosomal proteins plays a key role in ACN-induced apoptosis and cell cycle modulation.

### 2.6. ACN Attenuates c-Myc Stability

**Figure 6:**
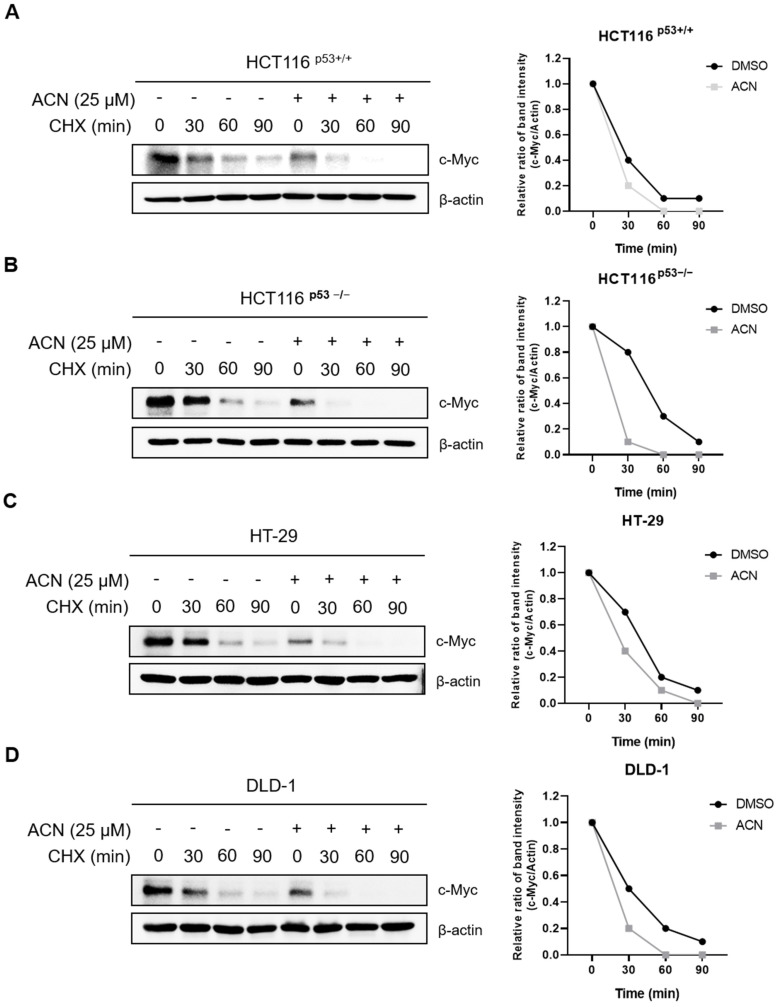
ACN inhibits c-Myc stability. Effect of CHX on c-Myc in HCT116^p53+/+^ (**A**), HCT116^p53−/−^ (**B**), HT-29 (**C**), and DLD-1 (**D**).

To investigate the effect of ACN on c-Myc stability, a CHX chase analysis was performed. ACN decreased the half-life of c-Myc compared with that in the control group (Figure 6). These results indicate that ACN regulates the half-life of c-Myc and reduces its expression.

### 2.7. ACN Regulates Serum-Induced c-Myc Stimulation

**Figure 7:**
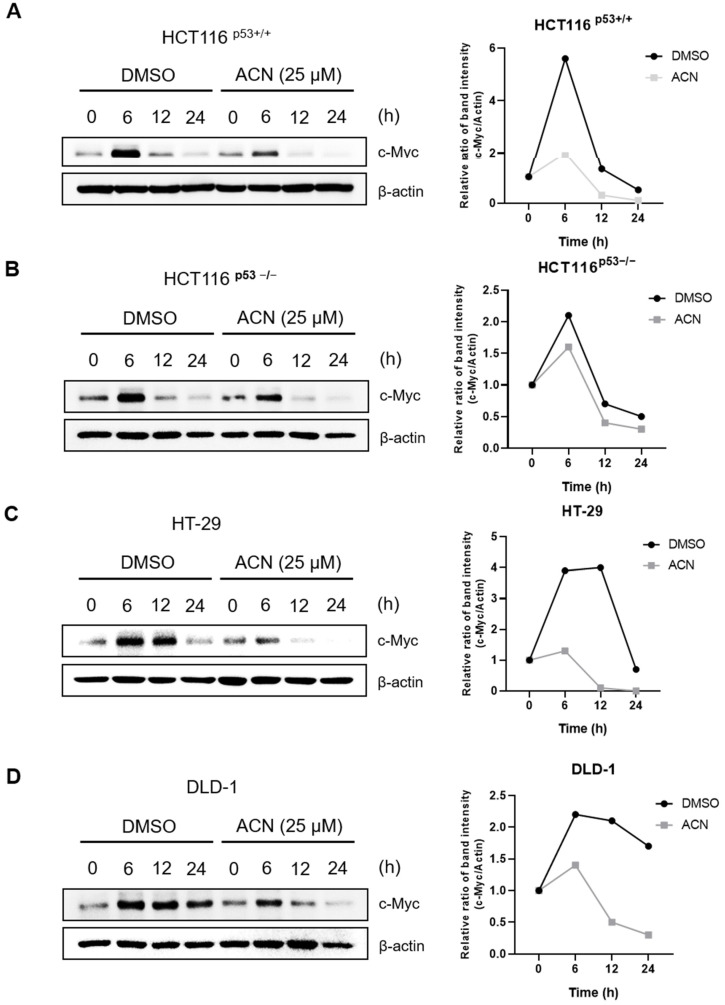
Expression of c-Myc via serum stimulation. Regulation of serum-responsive induction of c-Myc by ACN in HCT116^p53+/+^ (**A**), HCT116^p53−/−^ (**B**), HT-29 (**C**), and DLD-1 (**D**).

c-Myc expression changes rapidly during serum stimulation. To determine whether ACN affected serum-induced c-Myc stimulation, we compared c-Myc expression after treatment with ACN or DMSO via serum stimulation (Figure 7). This suggests that ACN rapidly regulates c-Myc expression following serum stimulation.

### 2.8. Potential Effect of ACN with 5-FU or Dox in Colon Cancer Cells

**Figure 8:**
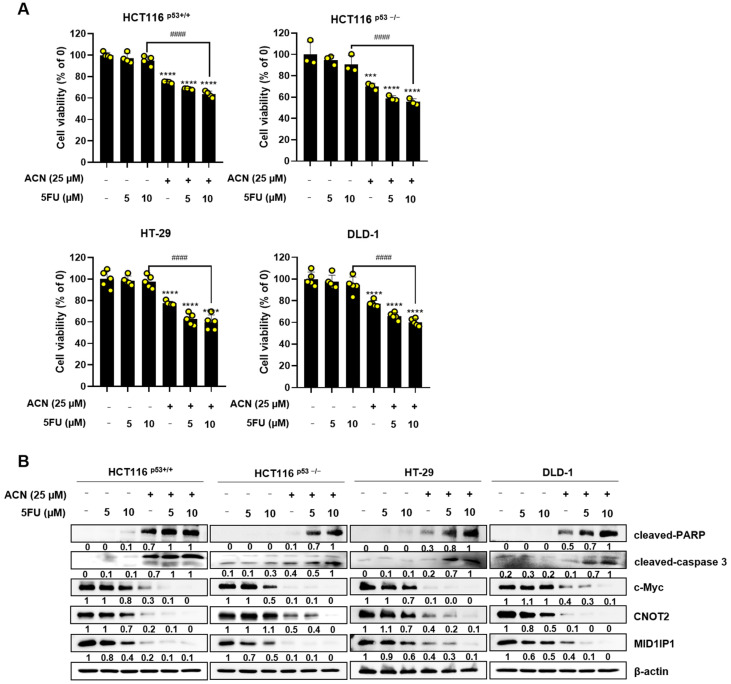

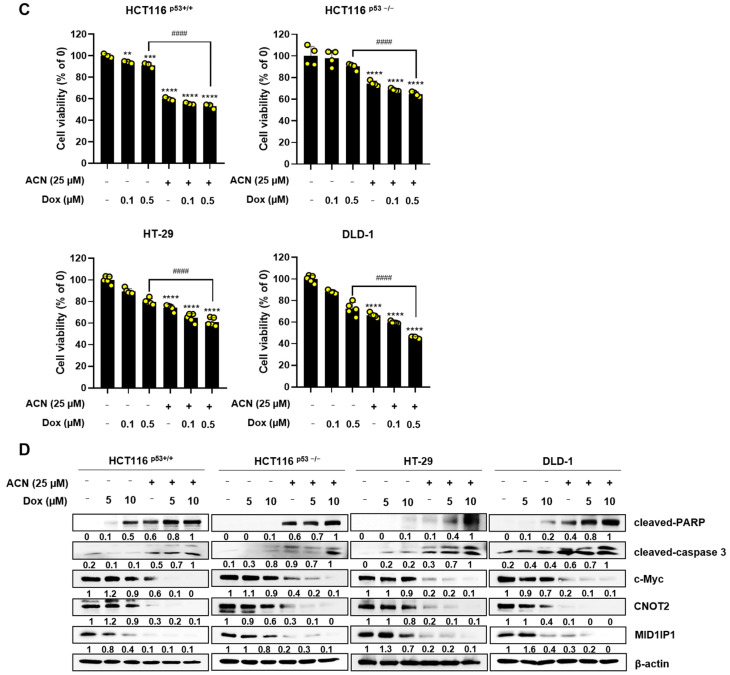
Combination effect of ACN with 5-FU or Dox. Combined effect of ACN and 5-FU on cell viability (**A**) and protein expression (**B**). Combined effect of ACN and Dox on cell viability (**C**) and protein expression (**D**). Data (n ≥ 3) are represented as the mean ± SD. ** *p* <0.01, *** *p* < 0.001, and **** *p* < 0.0001.

Minimizing side effects by reducing the dose of the anticancer drugs 5-FU and Dox is important. Therefore, we investigated whether the combined effect of ACN could maximize its efficacy using low-dose 5-FU and Dox. The combined treatment significantly reduced cell viability compared to that in the control group (Figure 8A,C). The combination of anticancer drugs and ACN alleviated c-Myc, MID1IP1, and CNOT2 expression, and increased the expression of cleaved PARP and cleaved caspase3 compared to the control group (Figure 8B,D). In conclusion, ACN enhances the combined effect of anticancer drugs in colon cancer cells. 

## 3. Discussion

Despite the development of new treatments for CRC, side effects and drug resistance continue to emerge. Therefore, it is crucial to discover novel natural compounds that can demonstrate a synergistic effect while minimizing these issues.

Corydalis species have been utilized in traditional Chinese medicine for pain relief, renowned for their anti-inflammatory and anticancer properties. ACN, a major alkaloid extracted from *C. ambigua Cham. et Schltdl* tubers, demonstrates efficacy in treating Parkinson’s disease [16] and is anti-inflammatory [17]. However, the molecular mechanisms and anticancer activity of *C. ambigua Cham. et Schltdl*-derived ACN remain unclear. In this study, we confirmed that ACN induces apoptosis by negatively regulating oncogenes, including c-Myc, CNOT2, and MID1IP1, in various colon cancer cells.

To determine the concentration of ACN with therapeutic effects in human colorectal cancer cells HCT116^+/+^, HCT^−/−^, HT-29, and DLD-1, while maintaining non-toxicity in normal colon cells, we treated each concentration of ACN. As a result, the treatment of ACN up to 100 μM led to a notable reduction in the viability of cancer cells, while demonstrating no significant toxicity in normal cells. Therefore, additional experiments were performed up to 100 μM. 

We demonstrated that ACN inhibited cell viability and proliferation in a dose-dependent manner. Our study showed that ACN rescued cell viability from apoptosis inhibitor, indicating a potential protective role of ACN against apoptosis while simultaneously increasing cleaved PARP and cleaved caspase3. Furthermore, ACN inhibits the protein expression of cyclin D1, CDK4, and CDK2, which play roles in regulating cell cycle progression. The downregulation of cell cycle-related proteins suggests the possibility of cell cycle arrest, further contributing to the inhibitory effect of can on colon cancer cell growth. In addition, we observed an increase in the cell population in the G2/M phase. In this studcanACN induced apoptosis and cell cycle arrest in colon cancer cells.

c-Myc is deregulated and highly expressed in human cancers, including colon cancer [26]. Excessive expression of c-Myc can result in DNA damage, subsequently initiating apoptosis through the activation of DNA damage response pathways [27]. Therefore, the use of c-Myc as a therapeutic strategy is attractive for cancer therapy [28]. CNOT2 is associated with MID1IP1 in the regulation of c-Myc expression and the induction of apoptosis in cancer cells. MID1IP1 is regulated by the co-localization of c-Myc, which is mediated by the ribosomal proteins L5, L11, and CNOT2 [6]. In this study, we observedcanat ACN is a potential target for anticancer activity via c-Myc regulation. Interestingly, ACN reduced the expression of MID1IP1 and CNOT2 and further increased the expression of cleaved PARP and cleaved caspase3, which are related to apoptosis in colon cancer cells when knocked down, respectively, with ACN. Additionally, ACN quickly regulated the expression of c-Myc induced by serum stimulation. We demonstrated that ACN inhibits c-Myc via CNOT2 or MID1IP1. 

The commonly used chemotherapeutic drugs, 5-FU and Dox, have dose-limiting toxicity and drug resistance; therefore, a new adjuvant treatment must be developed to overcome these limitations. We observed a significant decrease in cell viability when ACN was used in combination with low doses of 5-FU and Dox. Combination treatment also increased apoptosis and further reduced expression of the oncogenes c-Myc, CNOT2, and MID1IP1 in colon cancer cells. This suggests that ACN can be used in combination with low-dose 5-FU and Dox to minimize the side effects of chemotherapy and maximize its therapeutic effect. 

In conclusion, this study demonstrated the anticancer activity of ACN in colon cancer cells. ACN induces apoptosis and cell cycle arrest to inhibit cancer cell growth by inhibiting the c-Myc signaling pathway. These results suggest that ACN can be used for chemotherapy with low-dose 5-FU and Dox.

We have confirmed that the anticancer effect of ACN is enhanced when combined with Dox or 5-FU. These results position ACN as a promising candidate as an anticancer drug in colon cancer cells, indicating its potential used in conjunction with existing treatments. However, we propose the imperative need for a comprehensive exploration of additional mechanisms regarding the synergistic effects with drugs currently undergoing clinical trials. Furthermore, a subsequent inquiry is in the planning stages to further investigate the anticancer mechanism of ACN in CRC. This includes specific exploration of whether ACN directly interacts with CNOT2 and MID1IP1, thereby influencing their expression and apoptosis.

## 4. Materials and Methods

### 4.1. Chemicals and Reagents

ACN was purchased from Chemfaces (Wuhan Chemfaces Biochemical Co., Ltd., Wuhan, China). Z-VAD-FMK (HY-15763) and 3-methyladenine (HY-19312) were purchased from MedChemExpress (Monmouth Junction, NJ, USA). Additionally, 5-FU was purchased from Sigma-Aldrich (St. Louis, MO, USA), and Dox was purchased from Selleck Chem (Munich, Germany). 

### 4.2. Cell Culture

CCD-18co, HCT116^p53+/+^, HCT116^p53−/−^, HT-29, and DLD-1 cells were purchased from the Korean Cell Line Bank (KCLB; Seoul, Republic of Korea). The cell lines were cultured at the Roswell Park Memorial Institute (RPMI)-1640 and supplemented with 1% antibiotics and 10% fetal bovine serum (FBS) in a 5% CO_2_ incubator at 37 °C.

### 4.3. Cytotoxicity Assay

Cells were distributed in 96-well plates (1 × 10^4^ cells/well) and treated with different concentrations of ACN for 24 h. Cytotoxicity was assessed using the 3-(4,5-dimethylthiazol-2-yl)-2,5-diphenyltetrazolium bromide (MTT; Sigma Aldrich Co., St. Louis, MO, USA) assay, and formazan was detected at 570 nm using a microplate reader (Bio-Rad, Hercules, CA, USA).

### 4.4. Colony Formation Assay

Cells were treated with ACN and each concentration was distributed in six-well plates (1 × 10^3^ cells/well), which were cultivated for 1 week at 5% CO_2_ and 37 °C. After colony formation, the cells were fixed for 10 min and stained using the Diff-Quick Kit (Sysmex Corporation, Kobe, Hyogo, Japan). 

### 4.5. Western Blotting

Immunoblotting was performed as described previously [29]. Briefly, ACN-treated cells (1 × 10^4^ cells/well) were lysed in a lysis buffer (Cell Signaling Technology, Beverly, MA, USA). Protein samples were separated using sodium dodecyl sulfate-polyacrylamide gel electrophoresis and transferred to nitrocellulose membranes. The antibodies used were as follows: cleaved PARP, cleaved caspase 3, CNOT2 (Cell Signaling Technology, Beverly, MA, USA), MID1IP1 (ProteinTech Antibody Group, Chicago, IL, USA), c-Myc (Abcam, Cambridge, UK), and β-actin (cat. no. sc-47778). These antibodies were diluted in tris-buffered saline + 0.1% Tween 20 (TBST; 1:1000) and incubated at 4 °C overnight. The horseradish peroxidase (HRP)-conjugated secondary antibodies (cat. sc-516102 and sc-2004) were incubated for 1 h and detected using an ImageQuant LAS 500 (GE Healthcare Life Sciences, Sydney, Australia).

### 4.6. Cell Cycle Analysis via Flow Cytometry

Cells were distributed in six-well plates (2 × 10^5^ cells/well) and treated with or without ACN. After being treated for 24 h, cells were washed twice with PBS, and fixed in 70% precooled ethanol for 3 h. Then, cells were resuspended in RNase A (100 µg/mL) and PI (50 µg/mL) for 20 min. The samples were analyzed using NovoCyte flow cytometery (ACEA Biosciences, San Diego, CA, USA).

### 4.7. Annexin V/Propidium Iodide (PI) Assay

The drug-treated cells (2 × 10^5^ cells/well) were harvested using trypsin and washed with phosphate-buffered saline (PBS). Subsequently, the cells were suspended in a binding buffer containing fluorescein isothiocyanate (FITC)-tagged annexin V and PI for 15 min. A FACSCanto II flow cytometery (BD Biosciences, Becton-Dickinson, Franklin Lakes, NJ, USA) was used to analyze apoptosis.

### 4.8. Cycloheximide (CHX) Chase Assay for c-Myc Stability

Cells were distributed in six-well plates (2 × 10^5^ cells/well) and exposed to 25 µM of ACN. Then, the protein synthesis inhibitor CHX (50 µg/mL) was added for 0, 30, 60, and 90 min. Western blotting was performed to confirm the expression of c-Myc and β-actin.

### 4.9. Serum Stimulation for c-Myc Induction

Cells were distributed in six-well plates (2 × 10^5^ cells/well) and starved in a serum-free medium for 24 h. Subsequently, 25 µM of ACN diluted in 20% FBS was incubated, and cells were harvested at 0, 6, 12, and 24 h. c-Myc and β-actin protein expression levels were measured using Western blotting.

### 4.10. Immunofluorescence Assay

Cells were distributed in two-well culture slides (1 × 10^5^ cells/well) and treated with or without ACN for 24 h. Cells were fixed with 4% paraformaldehyde for 15 min and permeabilized with 0.2% Triton X-100 at room temperature. An antibody against c-Myc (1:200; Abcam Cambridge, UK) was incubated overnight at 4 °C, then incubated with Alexa Fluor 488 goat anti-rabbit IgG antibody (1:500; Invitrogen, Waltham, MA, USA) for 1 h. Next, 4,6-diamidino-2-phenylindole (DAPI) was used for staining nuclei and observed using CELENATM S Digital Imaging System (Logos Biosystems, Inc., Anyang-si, Gyeonggi-do, Republic of Korea).

### 4.11. Gene Silencing Using Small Interfering RNA (siRNA)

Cells were seeded in six-well plates (7 × 10^4^ cells/well) and transfected with the control, CNOT2, MID1IP1, RPL5, or RPL11 siRNAs (Bioneer, Daejeon, Republic of Korea) using INTERFERin (Polyplus-transfection SA, Illkirch-Graffenstaden, France), according to the manufacturer’s instructions. After 48 h, the protein levels were detected using Western blotting.

### 4.12. Real-Time Polymerase Chain Reaction (RT-PCR)

Total RNA was extracted using Hybrid-R^TM^ (product no. 301-101, GeneAll, Seoul, Republic of Korea). RNA was measured using a NanoDrop spectrophotometer (Thermo Fisher Scientific, Waltham, MA, USA). Total RNA was converted to cDNA using the Maxime RT premix (product no. 25082, iNtRON Biotechnology, Seongnam-si, Republic of Korea). Real-time PCR was conducted using an Applied Biosystems Step One System (Applied Biosystems, Foster City, CA, USA) with the Universal SYBR Green Master Mix (product no. 4367659, Applied Biosystems, Foster City, CA, USA). Quantification based on the relative expression of a target gene versus GAPDH gene (2^−∆∆Ct^) was performed to determine the level of mRNA expression. The c-Myc, CNOT2, and MID1IP1 primers were purchased from Bioneer (Daejeon, Republic of Korea).

### 4.13. Statistical Analysis

All data were repeated at least thrice and are expressed as the mean ± standard deviation (SD). Student’s *t*-test was performed to compare the two groups, and a one-way analysis of variance (ANOVA) followed by Dunnett’s test was performed for multiple groups. GraphPad Prism software (version 8.0; San Diego, CA, USA) was used to determine the statistical significance.

## Data Availability

All data presented in this study are available from the corresponding author upon reasonable request.
